# THE IMPACT OF TRAUMA AND POSTTRAUMATIC STRESS SYMPTOMS ON CAREGIVER WELL-BEING

**DOI:** 10.1093/geroni/igae098.3142

**Published:** 2024-12-31

**Authors:** Christina Hugenschmidt, Natasha Collier, Gretchen Brenes

**Affiliations:** Wake Forest University School of Medicine, Winston-Salem, North Carolina, United States; Atrium Health Wake Forest Baptist, Winston-Salem, North Carolina, United States; Wake Forest University School of Medicine, Winston-Salem, North Carolina, United States

## Abstract

There is a high level of variability in caregiver experiences and outcomes, and up to 50% of variance in caregiver well-being remains unexplained. We propose that exposure to traumatic events and experience of post-traumatic stress symptoms (PTSS) may be a previously unrecognized dimension of caregiver burden. We surveyed 25 caregivers of persons with dementia (PWD) to assess 1) lifetime exposure to traumatic events, 2) post-traumatic stress symptoms (PTSS) related to the caregiving experience, and 3) associations between trauma exposures and PTSS and caregiver burden, depression, and grief. Caregivers averaged 66.4 years old (range: 36-81) and had been caring for their loved one an average of 5.5 years (range: 1-23 years). Caregivers had different relationships with the care recipients (daughter: n=9, spouse/partner: n=9, son: n=4, sister: n=2, friend: n=1) and n=11 (44%) lived with the care recipient. Forty percent of the sample had experienced at least one adverse childhood experience (ACE) and 96% reported at least one trauma as an adult. Four participants (16%) stated that caregiving for their PWD or a caregiving-related event was the most stressful event of their life, and 35% had elevated PTSS related to caregiving. ACEs and PTSS in response to caregiving were highly predictive of important outcomes for caregiver well-being, including depression (ps ≤ 0.03), prolonged grief (ps ≤ 0.03), and caregiver strain (ps ≤ 0.02). These findings suggest that trauma experienced before and during caregiving and PTSS are significantly associated with caregiver well-being.

